# Acceptability of the routine use and collection of a generic patient reported outcome measure from the perspective of healthcare staff: a qualitative study

**DOI:** 10.1186/s41687-023-00617-4

**Published:** 2023-07-31

**Authors:** David A Snowdon, Velandai Srikanth, Richard Beare, Kate E Noeske, Elizabeth Le, Bridget O’Bree, Nadine E Andrew

**Affiliations:** 1 National Centre for Healthy Ageing, Melbourne, VIC Australia; 2 Peninsula Clinical School, Central Clinical School, Monash University, Melbourne, VIC Australia; 3 Academic Unit, Peninsula Health, Frankston, VIC Australia; 4 Developmental Imaging, Murdoch Children’s Research Institute, Melbourne, VIC Australia

**Keywords:** Patient reported outcome measures, Quality of life, Qualitative research

## Abstract

**Background:**

Patient-reported outcome measures (PROMs) provide a measure of self-perceived health status or health-related quality of life. They have been used to support provider-patient decisions, healthcare delivery, and value-based healthcare models. A barrier to routine collection of PROMs is the perception that PROMs lack clinical utility. As such, it is important to establish clinicians’ acceptability of the PROM prior to large-scale implementation. This study explored the acceptability of the routine use and collection of a generic PROM in healthcare services from the perspective of healthcare staff.

**Methods:**

Individual semi-structured interviews were completed from September 2020 to March 2021 with 26 staff from two multi-disciplinary community healthcare services in Melbourne, Australia. Interviews explored their experiences of using the EQ-5D-5L in their routine care. Interviews were recorded and transcribed verbatim. Data were analysed according to a framework approach, using inductive and deductive techniques.

**Results:**

Participants discussed the acceptability of the EQ-5D-5L with reference to four themes: practicalities of use; holistic nature; influence on client care; and influence on health service. Participants found the EQ-5D-5L quick and easy to administer, and appreciated that it measured multiple domains of health that were relevant to their clients’ care. They believed the EQ-5D-5L helped to identify client problems, and inform service delivery. They also reported features that were less acceptable, including a lack of item specificity to some healthcare disciplines. Participants reported the challenge of managing conflict between their assessment of the client’s health and the client’s perspective of their own health, leading some to question whether the client could provide an accurate reflection of their own health.

**Conclusions:**

The EQ-5D-5L has several features that healthcare staff viewed as acceptable for routine collection and use in healthcare. Training on the validity of the patient reported perspective and broadening the scope of PROMs collection beyond the EQ-5D-5L should be considered to facilitate large-scale implementation.

**Supplementary Information:**

The online version contains supplementary material available at 10.1186/s41687-023-00617-4.

## Background

Value-based healthcare is a healthcare delivery model that aims to improve population health and reduce costs [1, 2]. These aims are achieved by incentivising providers to maximise patient outcomes relative to the amount or type of care provided [1, 3]. A key feature of value-based healthcare are health outcomes considered meaningful to patients [3].

Patient-reported outcome measures (PROMs) provide a measure of self-perceived health status or health-related quality of life (HRQoL) [4]. PROMs can be classified as generic or specific to a disease or health condition [5]. They have been used to support provider-patient decisions in clinical care, improvements in healthcare delivery, and value-based healthcare models [6–10]. To support value-based healthcare it is crucial that PROMs are routinely collected within the majority of the population of interest to minimise bias [4, 11]. However, large-scale implementation of PROMs is difficult to achieve [12, 13].

A barrier to routine collection of PROMs by health professionals is their perception that PROMs lack clinical utility [12]. As such, it is important to establish health professionals’ acceptability of the PROM prior to large-scale implementation,[12, 14] where ‘acceptability’ refers to the extent to which the PROM is judged as suitable from a content and collection perspective to the professional [15]. The health professional may consider the pros and cons of the PROM, how the PROM fits within the organisational culture, the appropriateness of the PROM in their clinical practice, their overall satisfaction with the PROM, and/or their intent to continue using the PROM [15]. Understanding the acceptable, and less acceptable, features of a PROM will inform implementation strategies to support large-scale implementation of PROMs [12].

There are few examples of engaging health professionals in the process of selecting PROMs for routine collection. Examples of health professional engagement in this process have limited their involvement to surveying them on the current use of PROMs in their clinical practice [16, 17]. Neglecting to capture health professionals’ perceptions on the acceptability of the use of PROMs in their clinical practice, prior to implementation, may partially explain why completion rates have generally been low [13, 16, 17]. Also, capturing only the patient perspective on the acceptability of PROMs use in their clinical care has resulted in similarly low completion rates, highlighting that both perspectives are likely required when planning implementation of PROMs [12, 18].

The aim of our study was to explore the acceptability of the routine use and collection of a generic PROM in healthcare services from the perspective of healthcare staff.

## Methods

### Study design

Qualitative research methods using in-depth semi-structured interviews were chosen to explore the acceptability of a generic PROM in healthcare services from the perspective of healthcare staff. Ethics approval was obtained from the Peninsula Health Human Research Ethics Committee (LNR/66113/PH-2020).

### Setting

This study is part of a broader body of work undertaken by the National Centre for Healthy Ageing, Health Research Data Platform, a collaboration between Monash University and Peninsula Health in Victoria, Australia. Central to the Data Platform is the implementation and integration of a system for routine collection of PROMs across an entire healthcare organisation.

Peninsula Health is a publicly funded healthcare organisation in Melbourne, Australia that services over 300,000 people [19]. In 2019, Peninsula Health implemented the routine collection of the EQ-5D-5L at two of its multidisciplinary community healthcare services [20]. The EQ-5D-5L was implemented to provide a measure of service performance and to inform initiatives aimed at improving the quality of healthcare delivery (i.e. quality improvement) [20]. Clinicians were also encouraged to use EQ-5D-5L responses to inform their clinical decision making with individual clients (i.e. inform provider-patient decisions).

The EQ-5D-5L is a generic measure of HRQoL with potential to be routinely administered in healthcare services to support value-based healthcare [20–23]. It provides an indication of health status across five health domains (mobility, self-care, usual activities, pain/discomfort, and anxiety/depression) and a visual analogue scale (VAS) rating of overall health between 0 and 100 where 0 indicates worst possible health and 100 indicates best possible health [24, 25]. Responses for each domain include: no problems; slight problems; moderate; severe; and extreme problems [25]. For both the domain items and VAS the respondent is asked to rate their health ‘TODAY’. Domain responses provide both a descriptive profile of health-related quality of life and an overall index (i.e. weighted utility score) which can be used in economic evaluations [26]. The EQ-5D-5L was developed from the EQ-5D-3L, the original 3-level response version of the tool which was created by the EuroQol Group in the 1980s [25, 26]. The EQ-5D-5L has a lower ceiling effect and higher sensitivity than the original version of the tool, and is reliable and valid across many areas of healthcare [27, 28]. It is available in more than 150 languages and can be administered using multiple modes (e.g. electronic, telephone, paper-based) [26].

**Fig. 1:**
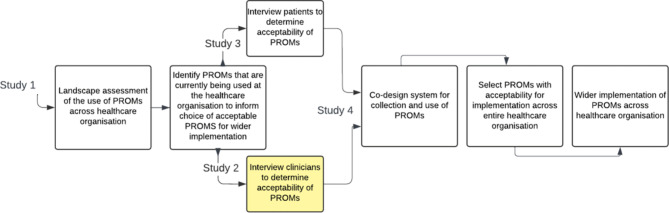
Planning implementation of routine collection of Patient Reported Outcome Measures across Peninsula Health

To expand PROMs collection across the entire organisation we proposed a program of work consisting of four studies (Fig. 1). These studies address the core steps involved in planning implementation of routine collection of PROMs by determining: purpose of collection; scope of the PROM; practical considerations of collection; patient and clinician acceptability of the PROM; and measurement properties of the PROM [12, 14, 29]. In this current study, we aimed to determine the acceptability of the EQ-5D-5L, the most commonly used PROM identified in Study 1, from the perspective of Peninsula Health staff.

### Participants

Staff were recruited from two multi-disciplinary community healthcare services that had implemented routine collection of the EQ-5D-5L. One was the community rehabilitation program; a short-term (i.e. 2–12 weeks) program that provides rehabilitation for clients who have undergone surgery, sustained an acute injury, and/or suffered an acute deterioration in health. The other was the community care program; a long-term program (i.e. 3–6 months), that provides care for people with chronic and complex health conditions. Clinicians, managers, healthcare assistants and administrative staff who were involved in the implementation and/or routine administration of the EQ-5D-5L were eligible to participate.

Eligible staff were invited to participate via email. All participants provided written informed consent.

### Data collection

Individual interviews were conducted via videoconferencing software by one researcher (KN) from September 2020 to March 2021. The interviewer had an allied health background and was experienced in conducting interviews. The interviewer did not have a relationship with participants. Participants were aware of the interviewer’s interest in the research topic.

The flexible interview guide was developed based on Bowen et al.’s feasibility framework, specifically the acceptability area of focus, and the Theoretical Domains Framework (Table 1) [15, 30, 31]. The guide was used to ensure consistency in gathering data from numerous participants and to ensure that this data were relevant to the construct of acceptability [30]. The guide was not used as a script; rather the interviewer was encouraged to be flexible and responsive to the interviewee, probing for further information where appropriate and covering the topics in the guide as dictated by the flow of the interview [30]. The interview guide was piloted with two clinicians; no changes were made to the guide following pilot testing.

**Table 1:** Semi-structured interview guide

Topic area	Sample questions
Introduction	Can you briefly describe the work that you do and the service that you provide to your clients/patients?
	What is your understanding of the EQ-5D-5L and patient reported quality of life outcomes? Are these important outcomes?
Implementation of EQ-5D-5 L	Can you briefly describe the changes that were made to your service?
	What was the ‘case’ for implementation of the EQ-5D-5L within the service?
	Did you perceive measurement of patient outcomes to be a problem in this service? Why?
	What was your role in implementing the EQ-5D-5L?
	How would you describe your experience of implementing EQ-5D-5L in the service?
	What worked well?
	What was difficult?
	To what extent did managers/peers/patients influence the implementation/use of the EQ-5D-5L?
Effect of EQ-5D-5 L on staff, work practices, and patient care	Now that the EQ-5D-5L is in place, how do you find it to work with?
	How does use of the EQ-5D-5L affect your workload?
	How sustainable is use of the EQ-5D-5L? How would you improve sustainability?
	What effect, if any, do you feel use of the EQ-5D-5L has (or will have) on patient care or outcomes?
Overall acceptability / future direction	What is your overall opinion of the EQ-5D-5L?
	Can you describe any other benefits or disadvantages from using the EQ-5D-5L?
	What are the incentives for using the EQ-5D-5L?
	Is the EQ-5D an appropriate outcome measure for your service to use?
	If you were to be in the position of overseeing the introduction of the EQ-5D-5L (or a similar outcome measure) in the future, is there anything you would do differently?
	How is the EQ-5D-5 L applicable to other areas of clinical care within Peninsula Health?

### Data analysis

Interview recordings were transcribed verbatim by a professional transcription service. Each participant was sent a copy of their transcript for clarification that the transcript conveyed what they intended to say.

Data were analysed according to a five-stage framework approach, using both inductive and deductive techniques [32]. Three authors familiarised themselves with the data and conducted a cursory inductive analysis of five transcripts each (DAS, EL, BOB) [32]. One author (DAS) developed an initial coding framework, which was reviewed by the analysis team. Two authors coded all data (DAS and EL). Data were charted to identify patterns in codes and form inductive themes. These themes and codes were then interpreted in the context of existing literature on the construct of acceptability, by three authors (DAS, EL, BOB) deductively mapping them to Sekhon et al.’s seven component constructs of acceptability for healthcare interventions (Table 2) [33, 34]. Authors initially mapped independently, then met to discuss and finalise the mapping process. All themes/codes were mapped to Sekhon et al.’s constructs of acceptability. The researchers felt that this inductive-deductive approach was optimal given our desire to better understand, interpret and contextualise how our inductive themes/codes explained the acceptable and less acceptable features of the EQ-5D-5L. Data were analysed using qualitative data management software [35].

**Table 2:** Sekhon et al.’s [34] seven component constructs of acceptability

Component construct	Description
Affective attitude	How an individual feels about an intervention.
Burden	The perceived amount of effort that is required to participate in the intervention.
Ethicality	The extent to which the intervention has good fit with an individual’s value system.
Intervention coherence	The extent to which the participant understands the intervention and how it works.
Opportunity costs	The extent to which benefits, profits or values must be given up to engage in the intervention.
Perceived effectiveness	The extent to which the intervention is perceived as likely to achieve its purpose.
Self-efficacy	The participant’s confidence that they can perform the behaviours required to participate in the intervention.

## Results

### Participants

Twenty-six interviews were conducted lasting between 32 and 73 min each, with approximately 23 h of data collected in total. Eleven participants (42%) were physiotherapists, four (15%) were occupational therapists, three (12%) were nurses, two were social workers (8%), one was a dietician (4%), two were speech pathologists (8%), one was a healthcare assistant (4%), two were administration staff (8%), and two (8%) were managers. The length of time that healthcare staff had been routinely collecting EQ-5D-5L data ranged from 12 to 24 months.

### Themes

Most participants believed that the EQ-5D-5L was acceptable for use with clients who receive community healthcare and stated intent to continue using the EQ-5D-5L. They reported the EQ-5D-5L was simple to use and provided a holistic measurement of clients’ HRQoL that could be used to guide patient care and quality improvement.

‘*It’s a good general patient reported outcome measure, which better aligns where the client sees their health to be, and where we think their health might be. So, we can tailor our program to better suit their needs.*’ (P13).

However, participants had concerns that the EQ-5D-5L did not capture all domains of health and therefore, may not be comprehensive enough to inform care for all clients.

‘*This tool is good for a big section of our program. But there are people for whom the Euroqol is probably not the best tool. It doesn’t gather enough for some client groups with cognitive and communication impairments.*’ (P1).

Participants discussed the acceptability of the EQ-5D-5L with reference to four main themes: practicalities of use; holistic nature; influence on client care; and influence on health service.

### Theme 1: practicalities of use – the EQ-5D-5L is quick and easy to administer

Participants reported that the EQ-5D-5L was quick and easy to administer; increasing its utility in their clinical practice. They were pleased it was relatively short compared to other PROMs and didn’t significantly redirect time from client care. Participants also appreciated that the EQ-5D-5L could be administered via telephone, and by administration staff or assistants. This flexibility saved clinicians time and assisted with completion rates.

‘*With all the discharges we were getting, the allied health assistants were able to administer those discharge Euroqols which was useful.*’ (P4).

However, the time involved in collating and analysing EQ-5D-5L data was viewed as a hinderance. Sophisticated information technology systems were seen as a potential solution.

‘*Human resource for inputting data into Excel, it takes a long time, and many different staff members, and many hours of their day doing that. I think using a piece of software to do it makes sense.*’ (P11).

Participants also reported that the EQ-5D-5L was suitable for most of their clients, who had no difficulty with completing the questionnaire with minimal instruction. There was some concern that clients may have been frustrated by the additional ‘paperwork’ but participants stated most were willing to complete the EQ-5D-5L once they were aware of its purpose.

‘*People think it’s yet another form. But if they have a good understanding that it’s for them and their measure of how they’re feeling, then they look at it differently.*’ (P15).

However, participants reported the EQ-5D-5L was less suitable for some clients, who had difficulty interpreting the VAS and could not quickly grasp the concept of rating their health on a scale from 0 to 100. Participants also reported that the EQ-5D-5L was less suitable for clients with hearing, communication and/or cognitive impairments, who had difficulty comprehending questions.

‘*Unless a person is at 100, I think sometimes it can take a little bit of time for a person to process where it is that they might be at in terms of that scale.*’ (P10).

### Theme 2: holistic nature – the EQ-5D-5L measures multiple aspects of health that are relevant across health disciplines

Participants viewed the EQ-5D-5L favourably because it provided a holistic measure of their clients’ health status and HRQoL. They identified that this aligned well with their own professional values and identified that these outcomes were important for community healthcare services to measure.

‘*Quality of life should be our main priority…Quality of life, at that point in their journey, where they are at home and they’re trying to readjust to ‘life as normal’, is really important.*’ (P9).

Many participants appreciated that each client’s opinion was unique, and valued that this enhanced their assessment by adding information that would otherwise not have been captured.

‘*It helps us gain greater information into what the client’s perspective of their condition is. Because a client’s perspective in terms of what their priority is, may be quite different to what a clinician might be*’ (P10).

Participants explained that the EQ-5D-5L was a good fit for multidisciplinary community healthcare services because it wasn’t discipline or condition specific, and measured multiple domains of health that were relevant to the client’s care.

‘*Rehabilitation isn’t just about one domain of someone’s health or quality of life. They might have significant mobility issues, that might impact on their level of anxiety. They’re all intertwined and it’s really important to capture different domains, which this does really well.*’ (P18).

However, participants acknowledged the EQ-5D-5L did not capture all domains of health that were relevant to their discipline (e.g. communication, cognition, diet). For example, they reported that the EQ-5D-5L did not contain items specific to communication and that this domain of health was only indirectly captured by the ‘usual activities’ item and VAS.

‘*I don’t think it is 100% comprehensive in covering all of the different disciplines and ways in which a person’s health might improve, although those things are encompassed within the overall health rating.*’ (P2).

Regardless, some participants believed that the EQ-5D-5 L may be more acceptable if used in conjunction with other discipline specific measures.

‘*(We should use) the Euroqol plus something more goal focussed. Like the Canadian Occupational Performance Measure, which is an Occupational Therapy tool.*’ (P1).

A few participants were concerned that any change in HRQoL was difficult to attribute to the program because these measures could be influenced by factors that were outside the control of the health service.

‘*I don’t feel that any change reflected in the scores is indicative of input…because it’s not what the program can change that would eventually lead them to conclude that attribution is from our program alone.*’ (P12).

### Theme 3: influence on client care – the EQ-5D-5L assists with identification of problems and informs care

Participants provided examples of how the EQ-5D-5L could influence the care they provided. They explained that it assisted in identification of problems, which they otherwise may have missed, and this helped them tailor their therapy to the client’s needs.

‘*It holds us accountable. If somebody’s marking on the admission Euroqol that they have severe anxiety and depression and I don’t do something about that, then I’m not doing my job.*’ (P9).

Participants reported that better identification of problems informed which disciplines they should refer the client to within the service. While identification of persistent problems at discharge helped to inform referral to other health services for ongoing care, and in some cases informed their decision to discharge the client from the service.

‘*If they tell me they don’t need any physio but they tick “I have moderate or severe difficulties with my mobility”. I’m like “Should I really be discharging? I probably should give you a call and just make sure it’s going okay.*” (P17).

Participants reported the challenge of managing conflict between their assessment of the client’s health and the client’s perspective of their own health. For some this was viewed as a positive outcome, as it provided insight into an aspect of the client’s health that they may have otherwise overlooked.

‘*(The EQ-5D-5L) gives me an indication of how they see themselves and what they think they are like. Then see if that matches my assessment or not, which helps me to think about all those certain areas that they need to work on, but didn’t come up when we discussed goals.*’ (P17).

For other participants this was viewed as a negative outcome; the conflict in perspectives led to them questioning the accuracy of the client’s perspective. The short recall period of the EQ-5D-5L (i.e. asking clients to rate their health ‘TODAY’) was also thought to impact on accuracy of client report, particularly for those with fluctuating health. In response to this conflict, participants sometimes found themselves possibly influencing participant responses through coercion to better fit their opinions of the client’s health.

‘*Sometimes the answers that the people gave didn’t reflect the situation the person was really in. They might have clear problems with their walking, then they might answer that they had a slight problem with walking around. And I would be thinking, well, you really aren’t walking much at all! I’m not quite sure how you’ve concluded that you’ve only got a slight problem. That was a little bit challenging, because it’s like, how much do I correct you about this?*’ (P16).

Participants explained that the EQ-5D-5L was used to provide feedback to clients on their progress during their time on the program. They believed that it was helpful to provide clients with an indication of how they had progressed, from their own perspective, and that highlighting how much clients had improved was beneficial for their wellbeing and motivation.

‘*Often they don’t remember rating themselves on their initial ones. So, giving them that feedback, they get a nice little boost knowing that they have improved.*’ (P4).

Participants also reported that feeding back EQ-5D-5L results to clients could be a negative experience for those who had deteriorated during their time on the program. As such, some clinicians were reluctant to administer the EQ-5D-5L to clients with progressive and/or terminal conditions, to avoid a potentially distressing discussion.

‘*If I know they have cancer or a life limiting disease, I’m less likely to do at the start. Because at the end it’s not going to be a good outcome.*’ (P11).

Few participants reported that the EQ-5D-5L did not influence their care. Those who felt it didn’t influence care believed that client problems were already captured with existing forms, or that the EQ-5D-5L did not provide the specificity of information on client impairment and activity limitation that is required to inform therapy.

‘*It gives a more holistic kind of view of the person’s health overall, relative to the more specifics of the discipline specific outcome measures. I’m not convinced that makes a clinically relevant difference to the person’s management.*’ (P2).

### Theme 4: influence on health service – the EQ-5D-5L provides a measure of service performance which can inform initiatives to improve care

Participants reported that because the EQ-5D-5L provided a holistic measure of health that was not specific to any healthcare discipline, it was an appropriate measure of service performance that could inform service delivery and structure. Most believed the EQ-5D-5L could be used to identify client populations who improve during their time on the program, and those who do not. They reported that this information could help identify trends that explain why clients do, or do not, improve and lead to new initiatives aimed at improving care.

‘*Hopefully we can figure out which patients did have good outcomes and perhaps look at trends on why. Hopefully that will lead to service changes and further research studies around, “If we did change this part of the program, was it successful?”* (P14).

For other participants it was more important for the EQ-5D-5L to demonstrate the benefit of the service; confirming that the service provides a high quality of care rather than identifying where they may improve their service.

‘*As a program as a whole, I don’t believe it influences our quality of care, but it may offer signals that we were on the right trajectory. Generally, Allied Health is delivering best practice but I think it just supports we are actually on the right track.*’ (P3).

Some participants were anxious that clients may not report improvement. They believed that there was potential for EQ-5D-5L data to be linked to funding and their own clinical performance. This made the EQ-5D-5L less attractive as they feared that there could be a negative effect on themselves and the service.

‘*If this tool ended up being linked to funding, which I know it’s not, but I’ve been around long enough to see things that aren’t supposed to be linked to funding, be used in that way. A tool biased towards particular outcomes, does it make it harder for people whose outcomes or needs are different. Does it end up creating any sort of bias in funding?* (P1)

Participants reported that some clinicians refused to administer the EQ-5D-5L to clients who they felt may not have improved enough to demonstrate the benefit of the program.

‘*I don’t know if they felt like they hadn’t done enough for the patient to show the improvement that they wanted to see. Sometimes, I’d ask about discharge Euroqols for certain clinicians’ clients, and it was like “Oh don’t worry, they wouldn’t have improved enough.“* (P6).

### Component constructs of acceptability

**Fig. 2:**
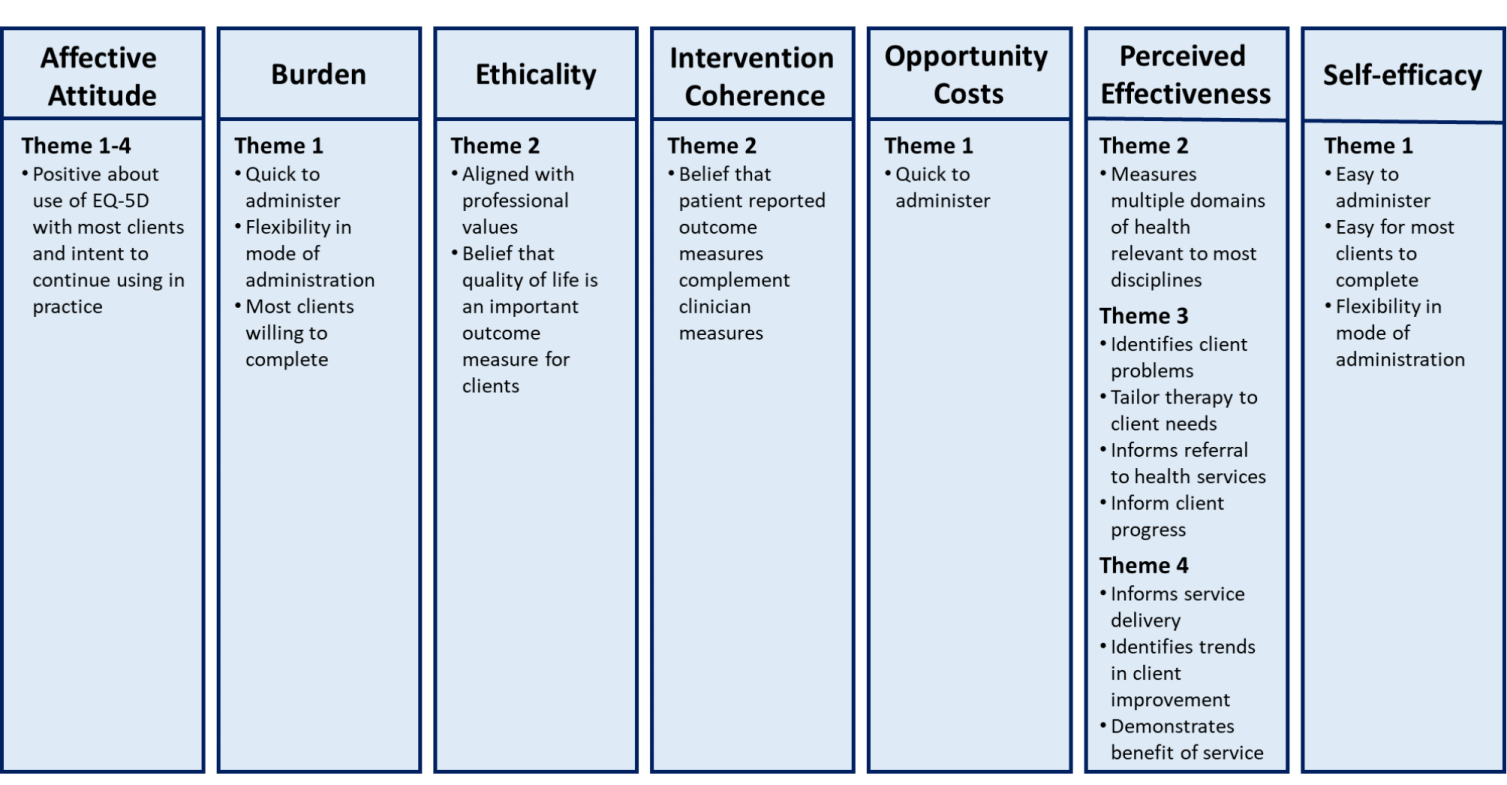
Findings related to the acceptable features of the EQ-5D-5L mapped to Sekhon et al.’s acceptability framework Legend: Theme 1 – Practicalities of use; Theme 2 – Holistic nature; Theme 3 – Influence on client care; Theme 4 – Influence on health service

**Fig. 3:**
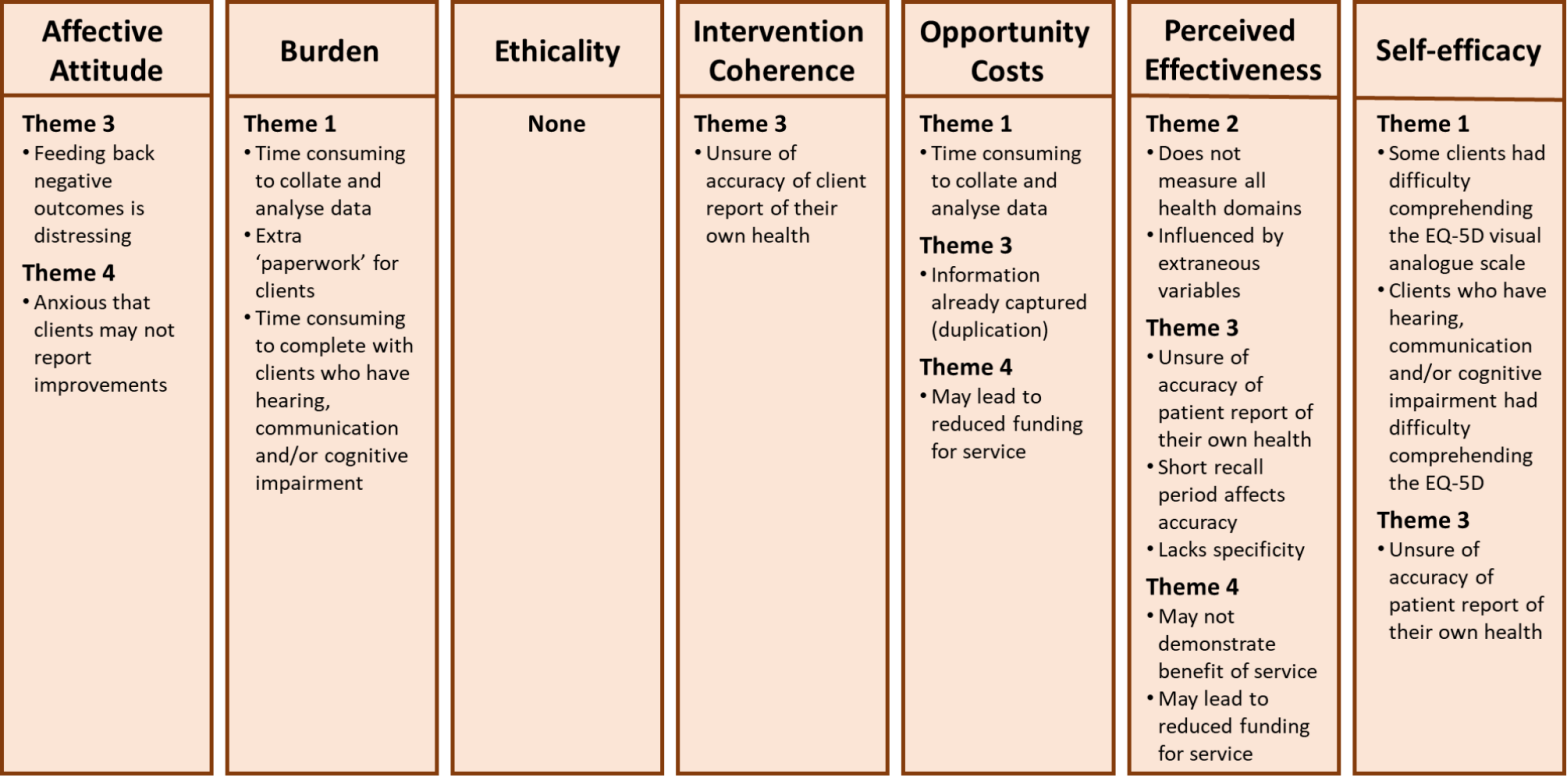
Findings related to less acceptable features of the EQ-5D-5L mapped to Sekhon et al.’s acceptability framework Legend: Theme 1 – Practicalities of use; Theme 2 – Holistic nature; Theme 3 – Influence on client care; Theme 4 – Influence on health service

Findings related to the acceptable features of the EQ-5D-5L were mapped to all seven component constructs of Sekhon et al.’s acceptability framework, with three or more themes contained within the component constructs of ‘affective attitude’ and ‘perceived effectiveness’ (Fig. 2). Findings related to less acceptable features of the EQ-5D-5L were mapped to six of the component constructs with three themes contained within the ‘perceived effectiveness’ and ‘opportunity costs’ component construct, while none were mapped to the ethicality component construct (Fig. 3).

## Discussion

Our results indicate that the EQ-5D-5L may be an acceptable measure for use in multidisciplinary healthcare settings, as healthcare staff expressed opinions indicating acceptability of the routine collection of a generic PROM within a health service. Acceptable features of the EQ-5D-5L were that it was quick and easy to administer; measured multiple aspects of health that were relevant across disciplines; had the potential to inform care through identification of client problems; and could be used to measure service performance. Less acceptable features were: its items lacked specificity to some disciplines; its short recall period (i.e. measuring health only ‘TODAY’); and concerns about the validity of PROMs in general (e.g. lack of trust in patient report).

Mapping to Sekhon et al.’s acceptability framework highlighted that many of the themes elicited in our study were related to both positive and negative attributes of the EQ-5D-5L with regards to ‘perceived effectiveness’. These results highlighted the contention between the positive attributes of a generic tool that is brief and broadly applicable to all conditions but at the expense of the level of detail often desired by clinicians from both a patient and discipline perspective. Previous research has shown that some clinicians find disease- or discipline-specific PROMs to be more valuable for informing and measuring clinical care outcomes than generic PROMS,[36] as generic PROMs may lack the specificity required to identify changes in health specific to the type of care provided [21, 29]. In particular, the EQ-5D-5L lacked items relating to cognition, communication and diet, important aspects of health for speech pathologists, psychologists and dietitians. One solution may be to include additional standalone items or measures of health domains that are relevant to these disciplines or clinical conditions [37]. However, for routine collection, consideration needs to be given to responder burden as this can impact on client acceptability and response rates [13]. To limit responder burden, where a condition specific PROM is used in conjunction with the EQ-5D-5L, the two measures could be mapped to identify and consolidate overlapping items [38]. This would allow data to be combined within the larger cohort without duplication of effort.

The items on the EQ-5D-5L have previously been shown to be easily understood by responders in healthcare settings [39]. Further, responders have reported less difficulty completing the VAS format questions than the Likert format questions [40–42]. Our finding that clinicians perceived some clients had difficulty with interpreting the VAS question conflicts with these previous findings. It is important that the clinicians’ perspective is validated against the clients’ perspective as this could create doubt over the validity of responses. If confirmed that clients have difficulty interpreting or completing the VAS, it will be important to codesign ways of supporting consumers to accurately complete the scale. Equally it will be important to educate clinicians that, as a measure of self-perceived health status, responses may not always agree with the clinician’s perception of the client’s health. Also, as a continuous measure the VAS will have greater variability in the range of responses than an ordinal measure such as the EQ-5D domain questions [43, 44].

Another feature of the EQ-5D-5L that was perceived as problematic in our study was the short recall period, which limited its usefulness for clients with fluctuating health. Most PROMs, including the EQ-5D-5L, do not capture health fluctuations unless administered frequently [45]. This has been shown to make it difficult to obtain valid client reports of health state or HRQoL in those who have fluctuating conditions [45, 46]. There is no simple solution to this problem. Although resource intensive, frequent self-administration of the EQ-5D-5L (i.e. multiple times in a day, across a number of days) is a valid method of measuring health state in people living with fluctuating conditions and may be a suitable option for some clients [45]. However, this approach may be difficult to implement within a framework designed for routine collection of PROMs.

The issue of clinicians questioning the accuracy of clients’ report of their own health is concerning and may demonstrate a lack of understanding of the underlying constructs of PROMs. When clinicians preference their own view of clients’ health status over that of the client, this is likely due to a belief that only the clinician possesses the expertise to make an accurate judgement of the client’s health, or that their opinion will be more objective than the client’s [47]. However, evidence shows that clinicians’ impression of clients’ health status and HRQoL is often inaccurate [47–49]. Further, the EQ-5D-5L is not a measure of function but rather a measure of health status, providing insights on how a client perceives their own health and how their health affects their HRQoL. This should be considered alongside clinicians’ assessment of health or function. Training and education on PROMs and the constructs that are being measured may help to address this challenge.

The purpose for using the EQ-5D-5L also appeared to affect its acceptability. In our study, the EQ-5D-5L was implemented for the primary purpose of quality improvement; to identify areas for improvement in service delivery [20]. Using PROMs to measure service performance has previously been identified as a concern for clinicians [12, 50]. In particular, they express that clients’ self-report of health state may be influenced by factors that are outside of their control and not accounted for when assessing performance [50]. While participants in our study agreed that a limitation of generic PROMs is that they can be influenced by many extraneous factors, most still found the EQ-5D-5L to be an acceptable measure of service performance. Participants who were opposed to the use of the EQ-5D-5L for measuring health service performance were mostly concerned about this information being linked to funding or performance management, rather than as a tool for identifying areas for quality improvement. This is an important consideration for services that intend to transition to value-based models of healthcare [3].

Our study included healthcare staff from a broad number of disciplines across two community health services, each with differing experiences in routine collection of the EQ-5D-5L, which enhances the generalisability of our results. The focused study aims, approach to data analysis, and team-based approach to data analysis satisfies the requirements for information power and improves the trustworthiness of our results [51]. However, there are also limitations that must be considered. Our study was conducted in an Australian healthcare organisation and results are only transferable to like settings. We also did not incorporate client perceptions of the acceptability of the EQ-5D-5L, whose insights should be considered alongside the perceptions of clinicians when determining whether the EQ-5D-5L is acceptable for wider implementation across a healthcare organisation [12]. Future work to this end is currently being undertaken by our research team.

## Conclusions

The routine collection of generic PROMs, in this case the EQ-5D-5L, within a health service was considered acceptable and useful by a range of professions across diverse patient groups. While this suggests that the EQ-5D-5L might be suitable for implementation across an entire healthcare organisation, the less acceptable features elicited from our results highlighted that further steps can be taken to maximise buy-in from clinicians. In particular, the scope of the collection system may need to be broadened beyond the EQ-5D-5L. Also, the belief that clients cannot accurately report on their health state may present a barrier to the routine use of PROMs across an organisation, creating biases in who is surveyed. Additional training and education on the underlying constructs of the PROM being used and the validity of the patient reported perspective should be considered as part of the implementation process.

## Electronic supplementary material

Below is the link to the electronic supplementary material.

Supplementary Material 1

## Data Availability

The data generated and analysed during the current study are not publicly available as participants did not provide consent for data sharing, however de-identified data is available from the corresponding author on reasonable request.

## Abbreviations

HRQoL: Health-related quality of life
PROM: Patient reported outcome measure
VAS: Visual analogue scale
